# Evolution of Anxiety Disorder Prevalence and Associated Factors in First Responders in Both the Medium and Long Terms after the January 2015 Terrorist Attacks in France

**DOI:** 10.1155/2023/5570808

**Published:** 2023-09-11

**Authors:** Cécile Girault, Lydéric Aubert, Yvon Motreff, Philippe Pirard, Cécile Vuillermoz, Stéphanie Vandentorren

**Affiliations:** ^1^Sorbonne Université, INSERM, Institut Pierre Louis d'Epidémiologie et de Santé Publique (IPLESP), Department of Social Epidemiology, F75012 Paris, France; ^2^Santé publique France, Direction des régions, cellule en region Antilles, F94415 Saint-Maurice, France; ^3^Santé publique France, Direction des maladies non transmissibles et traumatismes, F94415 Saint-Maurice, France; ^4^Santé publique France, Direction scientifique et internationale, F94415 Saint-Maurice, France; ^5^University of Bordeaux, INSERM, Bordeaux Population Health Research Center, U1219, F-33000 Bordeaux, France

## Abstract

First responders intervening in crisis situations are likely to subsequently develop mental disorders. We aimed to identify factors associated with anxiety disorders after a terrorist attack in both the medium and long terms. We used data collected on 180 first responders (medical/psychological health professionals and emergency rescue teams) interviewed face to face at 6-10 months (medium term) and 18-22 months (long term) after the January 2015 terrorist attacks in France. Anxiety disorders were measured using the Mini-International Neuropsychiatric Interview V6 and several other variables including terror exposure (comprising perceived level of exposure and real exposure level), sociodemographic characteristics, social support, mental health history, and access to psychological support resources. We developed a structural equation model to examine the interactions between these different factors. Postattack anxiety disorder prevalence in the medium and long terms was 16% and 14%, respectively. The main associated factors in the medium term were barriers to social support, perceived level of exposure, and a lack of psychological support resources. In the long term, the presence of anxiety disorders in the medium term and barriers to social support were directly associated with having anxiety disorders, while reexposure was indirectly associated. Barriers to social support played a crucial role in the prevalence of anxiety disorders in first responders following this traumatic event, both in the medium and long terms. Promoting stronger social cohesion and providing more psychological support resources following a disaster could help prevent anxiety disorders in this population.

## 1. Introduction

Terrorist attacks have the potential to induce trauma in individuals who are exposed to them. They can have long-term health implications such as physical disabilities, mental health disorders, and repercussions on work and social life [1]. Studies on psychiatric disorders developed by people exposed to terrorist attacks often investigate posttraumatic stress disorder (PTSD). Anxiety disorders are less frequently studied, despite being the most common category of psychiatric disorder [2]. Anxiety disorders may indeed have received less research attention compared to posttraumatic stress disorder (PTSD) for several reasons: (1) historically, in the DSM-IV, PTSD was categorized as an anxiety disorder, which may have overshadowed the specific study of other anxiety disorders [3]. (2) Symptoms of PTSD, such as flashbacks, nightmares, and hypervigilance, can be more readily observed and recognized compared to symptoms of certain anxiety disorders. Symptoms of anxiety disorders can be more elusive and challenging to detect, both by individuals themselves and by healthcare professionals [4].

Anxiety disorders are associated with other disorders, the most common being depression. Specifically, some studies show that over two-thirds of adults with an anxiety disorder also suffer from depression [5] and/or suicidal risk [6]. Another frequent comorbidity is attention deficit hyperactivity disorder (ADHD), with a lifetime estimated prevalence of 25% in adults and children with anxiety disorders [7]. Other comorbidities are bipolar disorder, substance dependence disorder, obsessive-compulsive disorder, and posttraumatic stress disorder [8], as well as medical conditions such as asthma [9] and hypertension [10]. Hypertension and anxiety may therefore be important predictors of future coronary heart disease [11].

Due to their higher risk of exposure to traumatic events, first responders are more susceptible to developing mental health disorders as a result of their interventions [12, 13]. A meta-analysis comparing the prevalence rates of posttraumatic stress disorder (PTSD) among first responders and the general population in various countries revealed that the worldwide pooled current prevalence of PTSD among first responders is estimated to be around 10%. [12]. In contrast, the 12-month prevalence rates of PTSD in the general population could be lower with 1.1% in Europe [14] and 3.5% in the USA [15]. Moreover, first responders are more likely than the general population to be exposed to terrorist attacks. It is therefore important to look at the development of mental health disorders in this population [12, 13]. In the literature, PTSD prevalence in first responders after terrorist attacks differs according to the location of the attack and the type of intervention group studied (e.g., differences between professionals and volunteers, or between different types of professionals) [12, 16, 17]. One study estimated the prevalence of mental disorders (PTSD, depression, and anxiety disorders) at 4% in police officers 5 to 12 months after they responded to the March 11, 2004 Madrid bombings [18], while another found a PTSD prevalence of 12% and 6% in firefighters and police officers, respectively, two years after the 9/11 New York attacks [19]. It is probable that the same diversity exists for anxiety disorders after terrorist attacks, but the literature on this subject is relatively scarce, including in the European context. Some studies indicated that first responders have a lower prevalence of mental health disorders compared to civilians who have been exposed to traumatic events [17, 20]. This could be attributed to factors such as their professional training, experience in handling stressful situations, and the development of coping mechanisms over time [19, 21]. However, it is important to consider potential biases that may influence the prevalence rates of disorders among responders. Reporting biases, including a better understanding of disorder measurement scales or a reluctance to report symptoms due to fear of stigma, could contribute to underestimate of prevalence of mental disorders [19]. Additionally, the healthy worker bias may play a role, as responders included in studies are often those who have continued working and are not on leave or have not changed jobs due to mental health concerns. Nevertheless, first responders are regularly exposed to potentially traumatic events. Finally, a recent literature review on the impact of terrorist attacks on the mental health of first responders indicated that questionnaires might tend to overestimate these prevalences, especially when the questionnaire is not specific to first responders [22]. Despite the limited literature on this topic, it is crucial to enhance our understanding of the psychological impact on workers and to further investigate the prevalence of mental health disorders in this population.

The factors most frequently associated with anxiety disorders in first responders after a terrorist attack are the type and level of exposure experienced during the event [17, 22]. However, the presence of mental health disorders after a traumatic event also depends on other factors, such as having been trained in traumatic events, education level, mental health history, and gender [13, 16, 22, 23]. Social support is widely recognized as a determinant of mental health [22, 24, 25]. A study highlighting the importance of investigating barriers to social support suggests that despite the presence of social support, individuals may face obstacles in actually accessing and receiving the support they need [26]. These barriers can negate or diminish the positive effect of social support on mental health outcomes [26, 27].

Most studies on first responders after a terrorist attack are cross-sectional in nature. Consequently, it is not possible to describe the evolution of anxiety disorder prevalence or the evolution of associated factors. One exception is a longitudinal study implemented after 9/11 which found that ten years after the attacks, 6% of first-responder police officers declared comorbid anxiety and PTSD, and 48% had comorbid major depressive disorder and anxiety disorder [28].

Furthermore, studies to date have not considered social factors, despite being strong determinants of mental health [29]. Finally, for categorical outcomes, epidemiological studies often use classic regression models which assume that explanatory factors are mutually independent. This choice means that the complexity of mental health and the interactions between associated factors cannot be examined.

In order to overcome some of these issues, the present paper aimed to assess the prevalence of anxiety disorders in both the medium and long terms in first responders to the January 2015 terrorist attacks in Paris and to examine associated factors.

## 2. Materials and Methods

### 2.1. Survey Design and Study Population

We used data from the IMPACTS survey (a French acronym for Investigation of Traumatic Manifestations of Post-Attack Trauma and Therapeutic Care and Support), which was conducted by *Santé Publique France,* the French national agency for public health, with the support from the Regional Health Agency (ARS) of the Paris area and the French National Institute of Health and Medical Research (Inserm). The survey is aimed at assessing the longitudinal mental health of civilians and first responders exposed to the January 2015 Paris attacks at 6-10 (i.e., medium term) and 18-22 (i.e., long term) months. It is also aimed at assessing the psychosomatic impact and the psychological support these persons received.

To be included, persons had to have been exposed during the first twelve hours following the attacks and meet at least one of the following conditions: (1) had been in touch by telephone with a person directly threatened or injured; (2) had taken care of a victim; (3) had provided medical/psychological support to a victim; (4) was a relative of a victim; (5) had returned afterward to the scene of the attack; (6) had been in direct contact with the terrorists (visual/auditory); (7) had watched video images of the event; and (8) had been a direct colleague (i.e., same unit) of a worker who died in the attacks. The different types of first responders were categorized into (i) firefighters from the Paris Fire Brigade, (ii) police officers and intervention forces, (iii) medical and medicopsychological emergency teams, and (iv) rescue workers (trained volunteers and professionals) from different organizations (French Red Cross, Civil Protection of Paris). The sampling process and survey methodology are detailed elsewhere [30].

The overall participation rate in the first wave was estimated at 65% (239/370). Indeed, 497 responded to our inclusion questionnaire, 127 did not meet eligibility criteria to be included, 131 refused to participate, and 239 agreed to an individual interview. The most frequently cited reason for refusal to participate to IMPACTS study was the lack of time (44%), not feeling concerned (26%), fear from confidential data and privacy (9%), and suffering still present or do not want to come back to a painful event (6%). Among those who agreed to respond (239), a total of 232 questionnaires were finally analyzed corresponding to 45 medical rescue workers (21 emergency medical staff and 24 emergency psychological staff), 60 firefighters from the Paris fire brigade, 55 policemen (including 16 intervention forces involved in the final assault), and 72 volunteer rescue workers (31 from Civil Protection of Paris and 41 from the French Red Cross). Our present study sample only investigated first responders who responded to both waves of the IMPACTS survey (*N* = 180).

The survey's self-developed questionnaires were administered face to face by trained clinical psychologists. The following data were collected: sociodemographics at the time of the study, experience of the attacks, perception of the attacks, experience of the consequences of the attacks, personal and professional situation before the events, postattack psychological support received, postattack social support received, and psychological thoughts and feelings at the time of the study.

### 2.2. Anxiety Disorders

Version 6 of the Mini-International Neuropsychiatric Interview (MINI) [31] was employed. It is a structured clinical interview which enables researchers to make diagnoses of psychiatric disorders according to DSM-IV or ICD-10 such as major depression, suicidal risk, panic disorder, agoraphobia, social phobia, posttraumatic stress disorder, alcohol dependence, alcohol abuse, substance-related disorders (nonalcohol), and generalized anxiety. The study outcome was having at least one of these four anxiety disorders (panic disorder, agoraphobia, social phobia, and generalized anxiety) (yes/no) in wave 1 (i.e., medium term) and/or in wave 2 (i.e., long term) of the survey.

### 2.3. Hypothesized Model of Associated Factors

**Figure 1:**
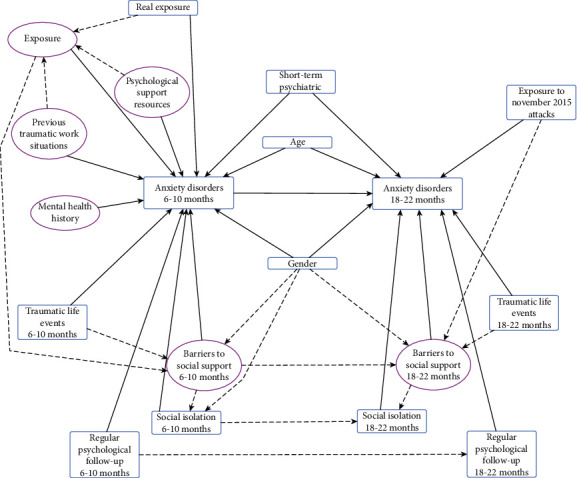
Hypothesized model of factors directly and indirectly associated with the presence of anxiety disorders in medium and long terms in first responders following the January 2015 attacks in Paris, France. Ellipses: latent variables; boxes: observed variables; straight lines: direct relationship; dotted lines: indirect relationship.

Taking into consideration the literature on anxiety disorders and PTSD following attacks and other traumatic events, we developed three main hypotheses (Figure 1) as follows: (i) the presence of anxiety disorders in the medium term was mainly related to the level of terror exposure (i.e., perceived and real) and to individual history (specific training received, work history, and mental health history [32]); (ii) the presence of anxiety disorders in the long term was related to the presence of anxiety disorders in the medium term and to reexposure to another terrorist attack between the two waves of the survey. We remind the reader that a second set of terrorist attacks occurred in Paris in November 2015 [33]; (iii) the presence of anxiety disorders was influenced by social isolation [26]. Any possible specific effect of this isolation may have been overshadowed by declared barriers to social support. In turn, these barriers may have negated any social support the participant declared he/she had received [26].

In addition to these three main hypotheses and based on available literature, we also hypothesized that gender [34], psychological support/follow-up [35], and personal history (i.e., psychological and work history) all influenced anxiety disorders [32].

### 2.4. Study Variables

#### 2.4.1. Latent Variables

*Terror exposure* was initially assessed using three variables in the first wave. The first was the perceived level of exposure, measured on a scale from 1 to 10, and recoded into three score modalities: 1-4, 5-7, and 8-10 according to the distribution and the sample size to represent low, moderate, and high perceived exposure. The second was the perception of having been targeted because of cultural, religious, or professional affiliation (yes/no). The third was the real level of exposure, assessed by recording the responder's physical proximity to the terrorists (meters, same street, adjacent building, etc.), recoded into three modalities: indirect witness, direct witness, and directly threatened.

*Barriers to social support* is a variable first constructed by researchers after terrorist attacks in Norway in 2011 [26]. It measures why first responders refrain from seeking help or support or from talking about their situation with others. It has five modalities as follows: (1) people were tired of hearing about the event; (2) people had enough problems of their own to deal with; (3) people would think the first responder was too caught up in what had happened; (4) the first responder would be putting too much burden on their friends; (5) people not present at the scene would not understand the first responder. This variable was collected in both of our study waves.

*Previous traumatic work situations* were based on two questions: (1) previous interventions as a first responder which had greatly affected the participant, and (2) whether the 2015 January attacks constituted the participant's first intervention in a disaster of this dimension. This variable was only collected in wave 1.

*Psychosocial support resources* were collected using three questions about specific training received in and access to psychological resources, as follows: psychosocial risk sensitization training (1), knowing a resource person within the professional environment (2), and having been sensitive to psychosocial risks (3). This variable was only collected in wave 1.

Finally, *mental health history* was collected by investigating the use of medication for specific disorders (sleep, anxiety, and depression) and psychological follow-up prior to the January 2015 attacks [36].

#### 2.4.2. Observed Variables

*Traumatic life events* were collected using a question on whether the participant had experienced other possible lifetime traumatic events (yes/no). It was collected in both waves; accordingly, it looked at one's life prior to the January 2015 attacks (wave 1) and the period between the two survey waves (second wave).

*Social isolation* was collected using a question about how alone or supported the participant felt. There were four modalities: very lonely, somewhat lonely, somewhat supported, and very supported [37]. These were then recoded into two modalities according to the distribution and the sample size (very supported versus all other modalities to observe the effect of strong social support). It was collected in both waves.

*Regular psychological follow-up* was collected with a question on whether the participant had engaged in regular care, support, or follow-up with a psychologist or with a psychiatrist since the January 2015 attacks (yes/no).

*Reexposure to attacks* was collected by asking participants whether they had also been exposed to the November 2015 attacks in Paris (yes/no). Naturally, this variable was only collected in wave 2.

Finally, *gender* and *age* were collected in both waves. Age was dichotomized into two modalities: <36 yo and ≥36 yo (corresponding to the median age of the sample).

### 2.5. Descriptive Analysis

The descriptive analysis was stratified by the type of first responder group (i.e., fire fighters, police officers and intervention forces) because the sociodemographic characteristics (age, gender, and educational level) were strongly and highly correlated with each group (Table 1).

### 2.6. Multivariate Analysis

First, to build the measurement model, we checked for correlations of the observed variables within each latent variable using Spearman's rank correlation. In this step, we excluded uncorrelated observed variables within the latent variables and redefined the latent variables based on the correlation results (some latent variables could be split in two). Second, we performed a scree-plot to check the monodimensionality of each latent construct. A scree-plot displays the eigenvalues associated with a component or factor in descending order versus the number of the component or factor. The point where the slope of the curve clearly levels off indicates the number of factors that need to be generated. Third, the hypothesized model of associated factors was validated using a confirmatory factor analysis (CFA) model, in order to test the relationship between observed variables and each latent variable. Observed variables which did not have a significant loading were excluded from the latent variable. Finally, we tested a structural equation model (SEM). To do this, we introduced the validated model of the CFA into the initial model of the SEM and then introduced the observed variables. We used a step-by-step approach, going backwards each time a model did not converge correctly. We stopped when all interactions had a *p* value <0.10. In order to obtain this robust model and these goodness-of-fit indices, we made choices that modified the initial model. Therefore, we chose to first remove the least significant relationships until we obtained a model for which all the relationships had a *p* value <0.10.

The estimator used in the CFA and SEM was the WSLMV (weighted least squares with mean and variance adjustment), which is recommended for variables that are dichotomous or categorical [38]. The coefficients were standardized and ranged from -1 (negative association) to 1 (positive association). The goodness-of-fit of the model was analyzed using four evaluation indices as follows: the Comparative Fit Index (CFI), the Tucker-Lewis Index (TLI) (≥0.90 is acceptable and ≥ 0.95 suggests a good fit for both), the root mean square error of approximation (RMSEA) (<0.06) and the standardized root mean square residual (SRMR) (<0.08).

## 3. Results

### 3.1. Study Population Characteristics

The study population (*n* = 180) was predominantly male (69%), 72% had an education level higher than high school diploma, and average age was 37 years (ranging from 19 to 70) (Table 1). A third of the respondents were rescue workers, a quarter firefighter, a fifth medical and medicopsychological emergency teams, and a quarter were police officers and intervention forces. Only eight persons were directly exposed during the attacks.

In comparison with the other intervention groups, medical and medicopsychological emergency teams were less likely to comprise males (54%), were on average older (44 years), and had the highest single exposure level (45% had indirect exposure). The firefighter group had the most men (86%) and was younger on average (33 years) than the other groups. Police officers and intervention forces were the category most exposed overall (33% had indirect exposure and 24% were directly threatened). This category was also slightly older (40 years on average) and had more women (36%) than the rescue worker and firefighter groups. Finally, rescue workers were the youngest (32 years old) and most educated (86% had at least a high school diploma) group. Together with firefighters, this group was the least exposed (32% had indirect exposure).

Anxiety disorder prevalence (i.e., having at least one of the four anxiety disorders studied) was 16% in wave 1 and 14% in wave 2. Agoraphobia was the most prevalent anxiety disorder in both waves (8% and 11%, respectively). It was also the only disorder with a higher prevalence in the second wave than in the first. Generalized anxiety disorder was the second most prevalent anxiety disorder (6% in wave 1 and 2% in wave 2).

### 3.2. Validation of Latent Constructs

At the validation step, as the “terror exposure” latent variable was not significant, we chose to split it. More specifically, we kept the two exposure perception variables (i.e., perceived exposure scale and perception of being targeted) in the latent variable and changed the real exposure variable (direct, indirect, etc.) to an observed variable.

All scree-plot curves levelled off above 1, indicating that each latent construct was unidimensional (see Figure S1 in the Supplementary Material).

### 3.3. Measurement Models

During the CFA model stage, we chose not to keep the “perceived exposure” latent variable (i.e., perceived exposure scale and perception of being targeted) because it prevented good convergence. At this point, we changed the perceived exposure scale to an observed variable (note: the perception of having been targeted was not retained). This was the only observed variable retained in the CFA model.

We chose not to individually keep the psychological disorder history variables (sleep, anxiety, depression, and psychological follow-up). Instead, we grouped them into one variable (i.e., having at least one of these items) as the CFA model showed better convergence.

The final CFA model fit the data well, with strong goodness-of-fit indices: CFI = 0.998; TLI = 0.998; RMSEA = 0.008 95%CI (0.000 − 0.043); and SRMR = 0.051. Finally, all factor loadings were significant at the 0.001 level (see Tables S2 in the Supplementary Material).

### 3.4. Final Model

**Figure 2:**
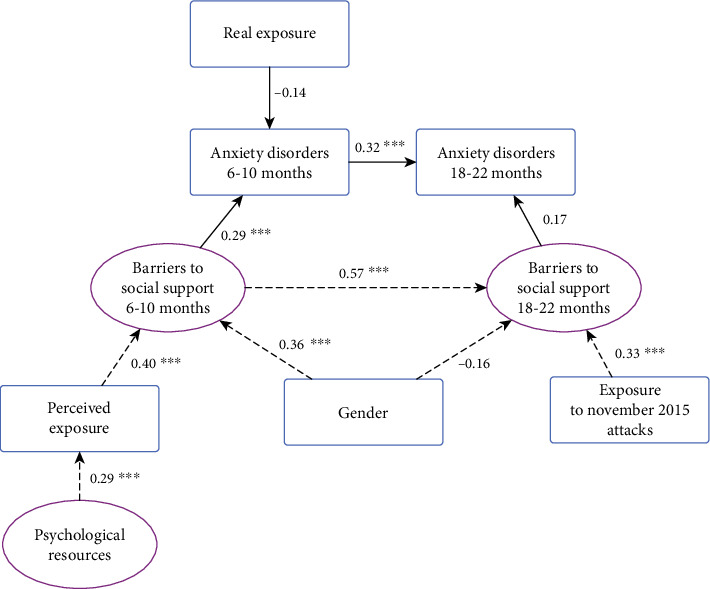
Final model of factors directly and indirectly associated with the presence of anxiety disorders in the medium and long terms in first responders following the January 2015 attacks in Paris, France. Ellipses: latent variables; boxes: observed variables; straight lines: direct relationship; dotted lines: indirect relationship; significance threshold: ⁣^∗∗∗^*p* ≤ 0.001; ⁣^∗∗^*p* ≤ 0.01; ⁣^∗^*p* ≤ 0.05.

In the final model (see Figure 2), the goodness-of-fit indices suggested good convergence and that the data fitted well: CFI = 0.973; TLI = 0.968; RMSEA = 0.024 95%CI (0.000 − 0.045) and SRMR = 0.065 (see Tables S3 in the Supplementary Material).

Finally, we only kept the “barriers to social support” and “psychological support resources” latent variables. In terms of observed variables, we retained the two remaining “terror exposure” variables (i.e., perceived exposure scale and real exposure), gender, and reexposure.

#### 3.4.1. Anxiety Disorders in the Medium Term

Barriers to social support were significantly positively associated with developing anxiety disorders (*β*_sd_ = 0.29; 95% CI (0.133;0.443); *p* < 0.001). Real exposure (indirect witness, direct witness, and directly threatened) seemed to be inversely related to developing anxiety disorders in the medium term (*β*_sd_ = −0.14; 95% CI (-0.291;0.009); *p* = 0.065), but this relationship was not significant at the 5% level.

A high perceived level of exposure was significantly associated with barriers to social support in the medium term (*β*_sd_ = 0.40; 95% CI (0.254;0.555); *p* < 0.001). Gender was also significantly associated with barriers to social support in the medium term. Women appeared to be at greater risk of presenting at least one anxiety disorder (*β*_sd_ = 0.36; 95% CI (0.196;0.519); *p* < 0.001). Finally, not having psychological support resources was positively associated with a high perceived level of exposure (*β*_sd_ = 0.29; 95% CI (0.0.86;0.503); *p* = 0.006).

#### 3.4.2. Anxiety Disorders over the Long Term

Having medium-term anxiety disorders was strongly correlated with having them in the long term (*β*_sd_ = 0.32; 95% CI (0.118;0.518); *p* = 0.002).

As with medium-term anxiety, barriers to social support seemed to be associated with the development of long-term anxiety disorders (*β*_sd_ = 0.17; 95% CI (-0.022;0.361); *p* = 0.083). However, unlike the medium-term context, this relationship was not significant at the 5% level.

Reexposure, that is to say also being exposed to the November 2015 attacks, was indirectly correlated with barriers to social support (*β*_sd_ = 0.33; 95% CI (0.180; 0.472); *p* < 0.001). The female gender was related with barriers to social support in the long term (*β*_sd_ = -0.16; 95% CI (-0.333;0.017); p = 0.077), but this relationship was not significant at the 5% level.

## 4. Discussion

Our study results indicate that the prevalence of anxiety disorders in a sample of first responders to the January 2015 Paris attacks was 16% and 14% over the medium (i.e., 6-10 months after the attacks) and long (i.e., 18-22 months) terms, respectively. These values are not very different from those found for the general French population in 2007 (a twelve-month prevalence of 15% [39]). Having said that, first responders are supposed to be better trained and prepared for such events. Moreover, the healthy worker effect [40] would lead us to believe that the prevalence would be much lower in a worker population than in the general population.

In the literature, PTSD prevalence varies according to the intervention group and the location of a disaster [12, 16, 17]. Studies on anxiety prevalence (disorders or symptoms) in first responders following a traumatic event also report differences according to the intervention group. For example, a 2018 study of firefighters and emergency medical technicians/paramedics who responded to emergency events in Arkansas, USA, found that 28% had moderate to severe anxiety symptoms [41]. Depending on the group to which they belong, first responders have different levels of training and experience in intervening in a traumatic event. The same is true in terms of training in psychosocial risks and support and follow-up (hierarchy, professional environment) received following an intervention. Furthermore, as we saw in the present study, different intervention groups have different socio-demographic characteristics (age, gender, level of education, etc.).

We could not include the “intervention group” variable into our final model because the non-ordinal categorical variable did not allow it.

### 4.1. Confirmation of Study Hypotheses

First, the presence of anxiety disorders in the medium term in our sample was mainly associated with the level of terror exposure (perceived and real) and individual history (specific training received, work history, and mental health history) [34]. Second, the presence of long-term anxiety disorders was quite strongly associated with medium-term anxiety disorders and exposure to the November 2015 terrorist attacks (which occurred between the two waves of our survey) [33]. Third, the presence of anxiety disorders was associated with a lack of social support [26]. However, any specific effect of the latter may have been overshadowed by declared barriers to social support. In turn, these barriers may have negated any social support the participant declared he/she had received [26].

#### 4.1.1. Anxiety Disorders in Both the Medium and Long Terms

According to our hypotheses, the presence of long-term anxiety disorders was strongly associated with the presence of medium-term anxiety disorders.

#### 4.1.2. Barriers to Social Support and Anxiety Disorders

Our study indicates that barriers to social support were associated with anxiety disorders. This result confirmed our third hypothesis that the presence of anxiety disorders was influenced by social isolation. However, any specific effect of the latter may have been overshadowed by declared barriers to social support. In turn, these barriers may have negated any social support the participant declared he/she had received. We found no effect of social isolation but a direct association with barriers to social support. The latter can be explained by the possibility that preventing oneself from speaking and using social support may increase the feeling of fear and the risk of developing anxiety disorders. However, agoraphobia and social phobia (two of the four anxiety disorders constituting our study outcome) are disorders that can prevent expression, speech, and contact with others; accordingly, it is possible that barriers are also an expression or a symptom of anxiety disorders.

The relationship between gender and barriers to social support in the second wave went in the opposite direction compared to the first wave: men had fewer barriers to social support than women. However, as this relationship was not significant (*p* value = 0.08) we could afford to ignore it.

#### 4.1.3. Indirect Association of Perceived Exposure and Anxiety Disorders Mediated by Barriers to Social Support

Our analysis supported our first hypothesis that anxiety disorders in the medium term are mainly related to the level of exposure. Specifically, we found an indirect association between a high perceived level of exposure and the presence of anxiety disorders. Perceived exposure was also associated with having barriers to social support in the medium term, while barriers to social support were associated with anxiety disorders. We also found an association between reexposure and barriers to social support in the long term. More specifically, having also been exposed to the November 2015 attacks in Paris was positively associated with barriers to social support.

These various results suggest that first responders who felt most exposed to the attacks or were exposed to the subsequent attacks faced more barriers (i.e., difficulties) to using social support. One possible explanation for this is that persons who were very exposed felt that they were not understood by those around them. First responders are also professionals that are socially represented as ‘strong' and therefore face a taboo on mental health, both because of the representation of their profession and perceived and experienced stigma. This taboo is very influential in the choice to use social support or not, as disclosing one's mental health problems may be seen as an admission of weakness [42]. For example, a study of police officers in a large Texas police department conducted between 2019 and 2020 showed four primary barriers to accessing mental health services, included the “stigma that officers who seek mental health services are not fit for duty” [43]. Another study showed that the most commonly cited barriers to accessing mental health care is a negative impact on one's career [44].

Interestingly, for the medium term, we observed an association between not having psychological support resources and a high perceived level of exposure. We can make the hypothesis that this was due to the perception of one's own level of vulnerability, which may be greater when there is a perceived lack of available psychological support resources.

The level of real exposure (i.e., physical proximity to the terrorists) was not significantly associated with the presence of medium-term anxiety disorders (*p* = 0.090).

#### 4.1.4. Indirect Association of Gender and Anxiety Disorders Mediated by Barriers to Social Support

We observed an indirect positive association between gender and the presence of anxiety disorders in the medium term. Being a woman was associated with barriers to social support, and barriers to social support were associated with anxiety disorders.

This indirect association was not significant in the long term.

#### 4.1.5. Associations Not Found

Our study failed to show a positive significant association between psychological support/follow-up and the presence of anxiety disorders even at the 10% threshold either in the medium or long terms. It is possible that two effects cancel each other out here: on the one hand, first responders who were going to or who had already consulted may have decided to do so because they had psychological disorders. On the other hand, having support or follow-up may have limited the development of the disorders.

Our study also failed to show any association between personal history (mental health history or work history) and anxiety disorders. Neither had these variables (work history and mental health history) any significant relationship with the other variables tested. This was a population that has more likely to have already responded to disasters and whose anxieties may be reactivated by reexposure, as shown by reexposure variable for those who also experienced the November 2015 attacks.

### 4.2. Study Limitations

This study had limitations. First, the relatively small sample did not allow us to test for more associations. This was particularly true for questions about previous history (psychological and trauma), work interruption, and drug use. Sample size can also be an issue when using the SEM method [45]. A minimum sample size of 100-150 individuals is recommended in some papers, while others suggest at least 200 individuals [46–48]. We chose to keep this model despite our small sample size because the final model has sufficient statistical power (0.975), and all the goodness-of-fit indices (TLI, CFI, RMSEA, and SRMR) have satisfactory thresholds [16]. Second, the final model comprised associations at the 10% significance threshold, and we removed some of these relationships. Accordingly, the indices were no longer as robust. Finally, selection bias is very probable, and it is likely that the study underestimated the true frequency of anxiety disorders because people with such disorders may have participated less in the survey. Due to these limitations, a confirmatory study would be helpful.

## 5. Conclusions

This study highlights the key role of barriers to social support in the development of anxiety disorders in both the medium and long terms in first responders after a terrorist attack and demonstrates that women, those who had a high perceived level of exposure, and those subsequently exposed to another attack all presented more barriers to social support. These results underline the importance of the need for specific resources for first responders (tailored training, psychological resource person, etc.) which could help them deal with exposure to traumatic events, and which could fight against the stigma of mental health. For example, one good practice might be to systematically offer support to combat stigma and the internalization of barriers. Prevention actions are also possible, for example, by screening for internalization barriers.

## Figures and Tables

**Table 1:** Description of sociodemographics, terror exposure, and anxiety disorders among first responders exposed to the January 2015 terrorist attacks in Paris, France, IMPACTS survey, 2015-2016.

	All respondents	Medical and medicopsychological emergency teams	Firefighters	Rescue workers	Police officers and intervention forces	*p* value^1^
	*N* = 180 *n* (%)	*N* = 38 (21%)	*N* = 49 (27%)	*N* = 59 (33%)	*N* = 33 (18%)	
Age (years)						
Mean (range)	37 (19-70)	44 (25-70)	35 (22-54)	32 (19-52)	40 (26-55)	
Gender						0.009
Man	124 (69)	20 (53)	42 (86)	40 (68)	21 (64)	
Woman	56 (31)	18 (47)	7 (14)	19 (32)	12 (36)	
Educational level						0.028
≤ High school diploma	50 (28)	12 (32)	16 (33)	8 (14)	13 (39)	
> High school diploma	130 (72)	26 (68)	33 (67)	51 (86)	20 (61)	
Level of exposure						<0.001
Indirect witness	109 (61)	21 (55)	33 (67)	40 (68)	14 (42)	
Direct witness	63 (35)	17 (45)	16 (33)	19 (32)	11 (33)	
Directly threatened	8 (4)	—	—	—	8 (24)	
Mental health (wave 1)^2^						
No anxiety disorder	147 (84)	28 (80)	42 (88)	49 (83)	27 (84)	0.800
At least one anxiety disorder	28 (16)	7 (20)	6 (12)	10 (17)	5 (16)	
Agoraphobia	14 (8)					
Social anxiety disorder	2 (1)					
General anxiety disorder	10 (6)					
Panic disorder	1 (1)					
Missing data	5	3	1	—	1	
Mental health (wave 2)						
No anxiety disorder	154 (86)	32 (84)	42 (86)	50 (85)	30 (91)	0.900
At least one anxiety disorder	26 (14)	6 (16)	7 (14)	9 (15)	3 (9)	
Agoraphobia	19 (11)					
Social anxiety disorder	1 (1)					
General anxiety disorder	4 (2)					
Panic disorder	—					

^1^Pearson's chi-squared test. ^2^Wave 1 refers to the first survey wave, and wave 2 refers to the second.

## Data Availability

The data that support the findings of this study are available from the French Public Health Agency (Santé Publique France), but restrictions apply to the availability of these data, which were used under license for the current study and are therefore not publicly available. Data are, however, available from the authors upon reasonable request and with permission of the French Public Health Agency.

## Supplementary Materials

Supplementary Materials For correlation measures and scree plots of the initial latent variables, see S1. For the statistical outputs of the final CFA model, see S2. For the statistical outputs of the final SEM model, see S3.
