# Supplementary material for: Evolution of Anxiety Disorder Prevalence and Associated Factors in First Responders in Both the Medium and Long Terms after the January 2015 Terrorist Attacks in France
Source: Depress Anxiety. 2023 Sep 11;2023:5570808. doi: 10.1155/2023/5570808 (PMC11921837; doi:10.1155/2023/5570808)
Supplement: Supplementary Materials — For correlation measures and scree plots of the initial latent variables, see S1. For the statistical outputs of the final CFA model, see S2. For the statistical outputs of the final SEM model, see S3. [file 5570808.f1.zip › Table_S1.docx]

# **Supplementary** **Material**

## **S1. Initial latent variables – Correlations measure**

#### **Perceived terror-exposure**


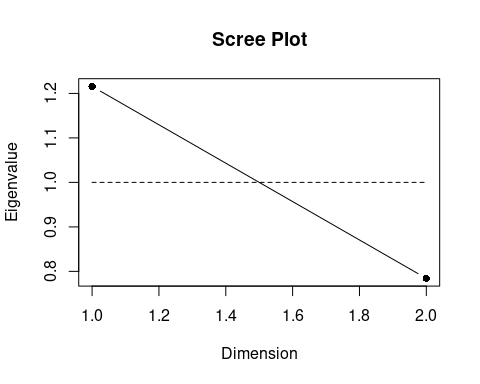


| VAR1 | VAR2 | RHO | PVALUE |
| --- | --- | --- | --- |
| iii_5_1_1_echelle_expo_sem2 | iii_5_1_1_echelle_expo_sem2 | 1.000 | 0 |
|  | iii_6_vict_appartenance | 0.219 | 0.0032 |
| iii_6_vict_appartenance | iii_5_1_1_echelle_expo_sem2 | 0.219 | 0.0032 |
|  | iii_6_vict_appartenance | 1.000 | 0 |

#### **Barrier to social support (medium-term)**


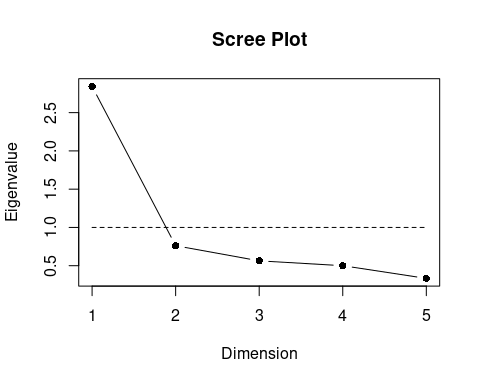


| VAR1 | VAR2 | RHO | PVALUE |
| --- | --- | --- | --- |
| retenu_soutien1_t1_sem | retenu_soutien1_t1_sem | 1.000 | 0 |
|  | retenu_soutien2_t1_sem | 0.616 | <0.001 |
|  | retenu_soutien3_t1_sem | 0.543 | <0.001 |
|  | retenu_soutien4_t1_sem | 0.336 | <0.001 |
|  | retenu_soutien5_t1_sem | 0.354 | <0.001 |
| retenu_soutien2_t1_sem | retenu_soutien1_t1_sem | 0.616 | <0.001 |
|  | retenu_soutien2_t1_sem | 1.000 | 0 |
|  | retenu_soutien3_t1_sem | 0.501 | <0.001 |
|  | retenu_soutien4_t1_sem | 0.488 | <0.001 |
|  | retenu_soutien5_t1_sem | 0.500 | <0.001 |
| retenu_soutien3_t1_sem | retenu_soutien1_t1_sem | 0.543 | <0.001 |
|  | retenu_soutien2_t1_sem | 0.501 | <0.001 |
|  | retenu_soutien3_t1_sem | 1.000 | 0 |
|  | retenu_soutien4_t1_sem | 0.412 | <0.001 |
|  | retenu_soutien5_t1_sem | 0.383 | <0.001 |
| retenu_soutien4_t1_sem | retenu_soutien1_t1_sem | 0.336 | <0.001 |
|  | retenu_soutien2_t1_sem | 0.488 | <0.001 |
|  | retenu_soutien3_t1_sem | 0.412 | <0.001 |
|  | retenu_soutien4_t1_sem | 1.000 | 0 |
|  | retenu_soutien5_t1_sem | 0.449 | <0.001 |
| retenu_soutien5_t1_sem | retenu_soutien1_t1_sem | 0.354 | <0.001 |
|  | retenu_soutien2_t1_sem | 0.500 | <0.001 |
|  | retenu_soutien3_t1_sem | 0.383 | <0.001 |
|  | retenu_soutien4_t1_sem | 0.449 | <0.001 |
|  | retenu_soutien5_t1_sem | 1.000 | 0 |

#### **Barrier to social support (long-term)**


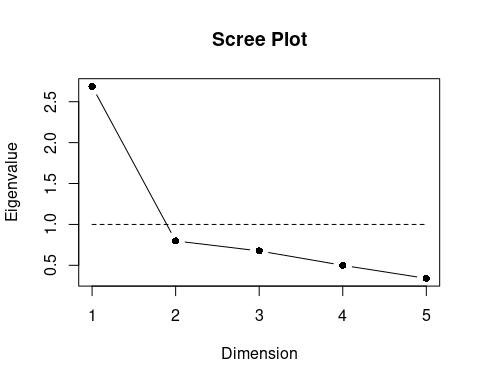


| VAR1 | VAR2 | RHO | PVALUE |
| --- | --- | --- | --- |
| q5_1.i1._sem | q5_1.i1._sem | 1.000 | 0 |
|  | q5_1.i2._sem | 0.656 | <0.001 |
|  | q5_1.i3._sem | 0.510 | <0.001 |
|  | q5_1.i4._sem | 0.468 | <0.001 |
|  | q5_1.i5._sem | 0.378 | <0.001 |
| q5_1.i2._sem | q5_1.i1._sem | 0.656 | <0.001 |
|  | q5_1.i2._sem | 1.000 | 0 |
|  | q5_1.i3._sem | 0.487 | <0.001 |
|  | q5_1.i4._sem | 0.465 | <0.001 |
|  | q5_1.i5._sem | 0.330 | <0.001 |
| q5_1.i3._sem | q5_1.i1._sem | 0.510 | <0.001 |
|  | q5_1.i2._sem | 0.487 | <0.001 |
|  | q5_1.i3._sem | 1.000 | 0 |
|  | q5_1.i4._sem | 0.325 | <0.001 |
|  | q5_1.i5._sem | 0.232 | 0.0017 |
| q5_1.i4._sem | q5_1.i1._sem | 0.468 | <0.001 |
|  | q5_1.i2._sem | 0.465 | <0.001 |
|  | q5_1.i3._sem | 0.325 | <0.001 |
|  | q5_1.i4._sem | 1.000 | 0 |
|  | q5_1.i5._sem | 0.257 | <0.001 |
| q5_1.i5._sem | q5_1.i1._sem | 0.378 | <0.001 |
|  | q5_1.i2._sem | 0.330 | <0.001 |
|  | q5_1.i3._sem | 0.232 | 0.0017 |
|  | q5_1.i4._sem | 0.257 | <0.001 |
|  | q5_1.i5._sem | 1.000 | 0 |

#### **Previous traumatic work situations**


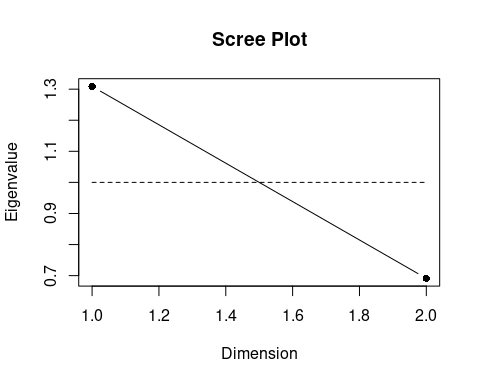


| VAR1 | VAR2 | RHO | PVALUE |
| --- | --- | --- | --- |
| v_2_5_interv_preced_eprouv_sem | v_2_5_interv_preced_eprouv_sem | 1.000 | 0 |
|  | v_2_6_1ere_interv_cata | 0.309 | <0.001 |
| v_2_6_1ere_interv_cata | v_2_5_interv_preced_eprouv_sem | 0.309 | <0.001 |
|  | v_2_6_1ere_interv_cata | 1.000 | 0 |

#### **Psychosocial resources**


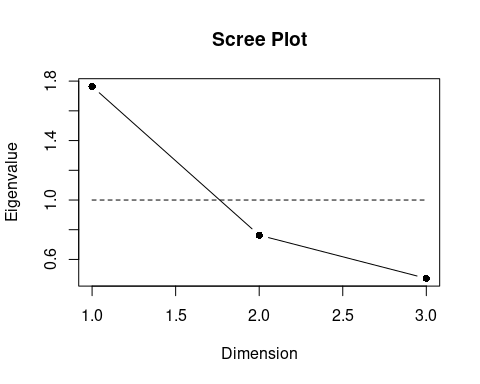


| VAR1 | VAR2 | RHO | PVALUE |
| --- | --- | --- | --- |
| v_2_2_personne_ressource_sem | v_2_2_personne_ressource_sem | 1.000 | 0 |
|  | v_2_3_form_stress_sem | 0.243 | 0.001 |
|  | v_2_1_sensib_risq_psy_sem | 0.395 | <0.001 |
| v_2_3_form_stress_sem | v_2_2_personne_ressource_sem | 0.243 | 0.001 |
|  | v_2_3_form_stress_sem | 1.000 | 0 |
|  | v_2_1_sensib_risq_psy_sem | 0.493 | <0.001 |
| v_2_1_sensib_risq_psy_sem | v_2_2_personne_ressource_sem | 0.395 | <0.001 |
|  | v_2_3_form_stress_sem | 0.493 | <0.001 |
|  | v_2_1_sensib_risq_psy_sem | 1.000 | 0 |

#### **Psychological history**


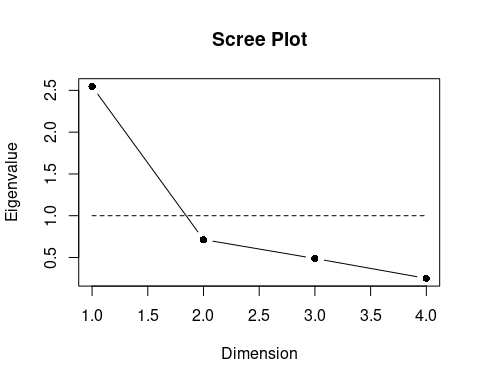


| VAR1 | VAR2 | RHO | PVALUE |
| --- | --- | --- | --- |
| v_1_1_1_medoc_sommeil | v_1_1_1_medoc_sommeil | 1.000 | 0 |
|  | v_1_1_2_medoc_angoisse | 0.602 | <0.001 |
|  | v_1_1_3_medoc_depression | 0.516 | <0.001 |
|  | v_1_2_suivi_psy_avt_evnmt | 0.351 | <0.001 |
| v_1_1_2_medoc_angoisse | v_1_1_1_medoc_sommeil | 0.602 | <0.001 |
|  | v_1_1_2_medoc_angoisse | 1.000 | 0 |
|  | v_1_1_3_medoc_depression | 0.711 | <0.001 |
|  | v_1_2_suivi_psy_avt_evnmt | 0.347 | <0.001 |
| v_1_1_3_medoc_depression | v_1_1_1_medoc_sommeil | 0.516 | <0.001 |
|  | v_1_1_2_medoc_angoisse | 0.711 | <0.001 |
|  | v_1_1_3_medoc_depression | 1.000 | 0 |
|  | v_1_2_suivi_psy_avt_evnmt | 0.513 | <0.001 |
| v_1_2_suivi_psy_avt_evnmt | v_1_1_1_medoc_sommeil | 0.351 | <0.001 |
|  | v_1_1_2_medoc_angoisse | 0.347 | <0.001 |
|  | v_1_1_3_medoc_depression | 0.513 | <0.001 |
|  | v_1_2_suivi_psy_avt_evnmt | 1.000 | 0 |

## 
