# Supplementary material for: Evolution of Anxiety Disorder Prevalence and Associated Factors in First Responders in Both the Medium and Long Terms after the January 2015 Terrorist Attacks in France
Source: Depress Anxiety. 2023 Sep 11;2023:5570808. doi: 10.1155/2023/5570808 (PMC11921837; doi:10.1155/2023/5570808)
Supplement: Supplementary Materials — For correlation measures and scree plots of the initial latent variables, see S1. For the statistical outputs of the final CFA model, see S2. For the statistical outputs of the final SEM model, see S3. [file 5570808.f1.zip › Table_S3.docx]

# **Supplementary** **Material**

## **S3. SEM** **analysis**

lavaan 0.6-11 ended normally after 87 iterations

| Estimator | DWLS |  |
| --- | --- | --- |
| Optimization method | NLMINB |  |
| Number of model parameters | 45 |  |
|  |  |  |
|  | Used | Total |
| Number of observations | 170 | 180 |

#### Model Test User Model

|  | Standard | Robust |
| --- | --- | --- |
| Test statistic | 121.777 | 159.392 |
| Degrees of freedom | 145 | 145 |
| P-value (Chi-square) | 0.920 | 0.196 |
| Scaling correction factor |  | 1.272 |
| Shift parameter |  | 63.683 |
| simple second-order correction | | |
|  |  |  |

#### Model Test Balseline Model

| Test statistic | 1285.650 | 694.933 |
| --- | --- | --- |
| Degrees of freedom | 168 | 168 |
| P-value (Chi-square) | 0.000 | 0.000 |
| Scaling correction factor |  | 2.121 |

User Model versus Baseline Model

| Comparative Fit Index (CFI) | 1.000 | 0.973 |
| --- | --- | --- |
| Tucker-Lewis Index (TLI) | 1.024 | 0.968 |
| Robust Comparative Fit Index (CFI) |  | NA |
| Robust Tucker-Lewis Index (TLI) |  | NA |

#### Root Mean Square Error of Approximation

| RMSEA | 0.000 | 0.024 |
| --- | --- | --- |
| 90% confidence interval - lower | 0.000 | 0.000 |
| 90% confidence interval - upper | 0.013 | 0.045 |
| P-value RMSEA <= 0.0 | 1.000 | 0.985 |
| Robust RMSEA |  | NA |
| 90% confidence interval - lower |  | 0.000 |
| 90% confidence interval - upper |  | NA |

#### Standardized Root Mean Square Residual

| SRMR | 0.065 | 0.065 |
| --- | --- | --- |

#### Parameter Estimates

| Standard errors | Robust.sem |
| --- | --- |
| Information | Expected |
| Information saturated (h1) model | Unstructured |

#### Latent variables

|  | Estimate | Std.Err | z-value | P(>\|z\|) | ci.lower | ci.upper | Std.lv | Std.all |
| --- | --- | --- | --- | --- | --- | --- | --- | --- |
| barrieresT1 =~ |  |  |  |  |  |  |  |  |
| rtn_stn1_t1_sm | 1.000 |  |  |  | 0.541 | 0.791 | 0.276 | 0.666 |
| rtn_stn2_t1_sm | 1.455 | 0.183 | 7.945 | 0.000 | 0.779 | 0.931 | 0.401 | 0.855 |
| rtn_stn3_t1_sm | 0.871 | 0.148 | 5.886 | 0.000 | 0.512 | 0.761 | 0.240 | 0.636 |
| rtn_stn4_t1_sm | 1.021 | 0.194 | 5.253 | 0.000 | 0.485 | 0.754 | 0.282 | 0.620 |
| rtn_stn5_t1_sm | 1.040 | 0.189 | 5.510 | 0.000 | 0.441 | 0.703 | 0.287 | 0.572 |
| barrieresT2 =~ |  |  |  |  |  |  |  |  |
| q5_1.i1._sem | 1.000 |  |  |  | 0.727 | 0.894 | 0.377 | 0.811 |
| q5_1.i2._sem | 0.925 | 0.074 | 12.575 | 0.000 | 0.649 | 0.860 | 0.349 | 0.754 |
| q5_1.i3._sem | 0.551 | 0.093 | 5.956 | 0.000 | 0.495 | 0.763 | 0.208 | 0.629 |
| q5_1.i4._sem | 0.676 | 0.096 | 7.003 | 0.000 | 0.393 | 0.687 | 0.254 | 0.540 |
| q5_1.i5._sem | 0.697 | 0.105 | 6.613 | 0.000 | 0.384 | 0.670 | 0.263 | 0.527 |
| bagage =~ |  |  |  |  |  |  |  |  |
| v_2_2_prsnn_r_ | 1.000 |  |  |  | 0.350 | 0.646 | 0.276 | 0.498 |
| v_2_3_frm_str_ | 1.455 | 0.183 | 7.945 | 0.000 | 0.388 | 0.679 | 0.401 | 0.533 |
| v_2_1_snsb_r__ | 0.871 | 0.148 | 5.886 | 0.000 | 0.704 | 0.958 | 0.240 | 0.881 |

#### Regressions

|  | Estimate | Std.Err | z-value | P(>\|z\|) | ci.lower | ci.upper | Std.lv | Std.all |
| --- | --- | --- | --- | --- | --- | --- | --- | --- |
| tb_anxieux ~ | | | | | | | | |
| barrieresT1 | 0.376 | 0.138 | 2.730 | 0.000 | 0.133 | 0.443 | 0.104 | 0.288 |
| algo_expo_sem | -0.089 | 0.050 | -1.786 | 0.065 | -0.291 | 0.009 | -0.089 | -0.141 |
| tb_anxieux_t2 ~ | | | | | | | | |
| tb_anxieux | 0.309 | 0.108 | 2.860 | 0.002 | 0.118 | 0.518 | 0.309 | 0.318 |
| barrieresT2 | 0.157 | 0.093 | 1.693 | 0.083 | -0.022 | 0.361 | 0.059 | 0.169 |
| barrieresT1 ~ | | | | | | | | |
| i_1_sexe | 0.213 | 0.061 | 3.495 | 0.000 | 0.196 | 0.519 | 0.771 | 0.357 |
| i_5_1_1_chl__2 | 0.134 | 0.031 | 4.371 | 0.000 | 0.254 | 0.555 | 0.484 | 0.404 |
| barrieresT2 ~ | | | | | | | | |
| barrieresT1 | 0.778 | 0.170 | 4.588 | 0.000 | 0.385 | 0.754 | 0.569 | 0.569 |
| i_1_sexe | -0.128 | 0.073 | -1.753 | 0.077 | -0.333 | 0.017 | -0.341 | -0.158 |
| atrsttntts_t2_ | 0.247 | 0.060 | 4.131 | 0.000 | 0.180 | 0.472 | 0.656 | 0.326 |
| iii_5_1_1_echelle_expo_sem2 ~ | | | | | | | | |
| bagage | 1.275 | 0.487 | 2.618 | 0.006 | 0.086 | 0.503 | 0.246 | 0.295 |

#### Covariances

|  | Estimate | Std.Err | | z-value | | P(>\|z\|) | | ci.lower | ci.upper | Std.lv | Std.all |
| --- | --- | --- | --- | --- | --- | --- | --- | --- | --- | --- | --- |
| algo_expo_sem ~~ | | |  | |  | |  |  |  |  |  |
| i_1_sexe | 0.008 | 0.026 | | 0.319 | | 0.749 | | -0.158 | 0.220 | 0.008 | 0.031 |
| atrsttntts_t2_ | -0.038 | 0.026 | | -1.475 | | 0.141 | | -0.310 | 0.044 | -0.038 | -0.133 |
| i_1_sexe ~~ |  |  | |  | |  | |  |  |  |  |
| atrsttntts_t2_ | 0.022 | 0.021 | | 1.047 | | 0.295 | | -0.083 | 0.273 | 0.022 | 0.095 |

#### Variances

|  | Estimate | Std.Err | z-value | P(>\|z\|) | ci.lower | ci.upper | Std.lv | Std.all |
| --- | --- | --- | --- | --- | --- | --- | --- | --- |
| .rtn_stn1_t1_sm | 0.095 | 0.014 | 6.800 | 0.000 | 0.068 | 0.123 | 0.095 | 0.556 |
| .rtn_stn2_t1_sm | 0.059 | 0.014 | 4.096 | 0.000 | 0.031 | 0.088 | 0.059 | 0.269 |
| .rtn_stn3_t1_sm | 0.085 | 0.012 | 7.251 | 0.000 | 0.062 | 0.108 | 0.085 | 0.595 |
| .rtn_stn4_t1_sm | 0.127 | 0.018 | 7.106 | 0.000 | 0.092 | 0.162 | 0.127 | 0.616 |
| .rtn_stn5_t1_sm | 0.169 | 0.019 | 8.785 | 0.000 | 0.131 | 0.207 | 0.169 | 0.673 |
| .q5_1.i1._sem | 0.074 | 0.015 | 4.806 | 0.000 | 0.044 | 0.104 | 0.074 | 0.343 |
| .q5_1.i2._sem | 0.092 | 0.018 | 5.112 | 0.000 | 0.057 | 0.127 | 0.092 | 0.431 |
| .q5_1.i3._sem | 0.066 | 0.010 | 6.458 | 0.000 | 0.046 | 0.086 | 0.066 | 0.604 |
| .q5_1.i4._sem | 0.157 | 0.020 | 8.052 | 0.000 | 0.119 | 0.196 | 0.157 | 0.709 |
| .q5_1.i5._sem | 0.179 | 0.019 | 9.268 | 0.000 | 0.141 | 0.217 | 0.179 | 0.722 |
| .v_2_2_prsnn_r_ | 0.113 | 0.015 | 7.743 | 0.000 | 0.084 | 0.141 | 0.113 | 0.752 |
| .v_2_3_frm_str_ | 0.156 | 0.018 | 8.851 | 0.000 | 0.121 | 0.191 | 0.156 | 0.715 |
| .v_2_1_snsb_r__ | 0.054 | 0.039 | 1.407 | 0.160 | -0.021 | 0.130 | 0.054 | 0.224 |
| .tb_anxieux | 0.117 | 0.017 | 7.023 | 0.000 | 0.084 | 0.149 | 0.117 | 0.898 |
| .tb_anxieux_t2 | 0.104 | 0.016 | 6.356 | 0.000 | 0.072 | 0.136 | 0.104 | 0.853 |
| .i_5_1_1_chl__2 | 0.637 | 0.055 | 11.565 | 0.000 | 0.529 | 0.744 | 0.637 | 0.913 |
| .barrieresT1 | 0.054 | 0.014 | 3.921 | 0.000 | 0.027 | 0.081 | 0.709 | 0.709 |
| .barrieresT2 | 0.086 | 0.017 | 5.114 | 0.000 | 0.053 | 0.119 | 0.606 | 0.606 |
| bagage | 0.037 | 0.014 | 2.713 | 0.007 | 0.010 | 0.064 | 1.000 | 1.000 |
| algo_expo_sem | 0.329 | 0.034 | 9.643 | 0.000 | 0.262 | 0.396 | 0.329 | 1.000 |
| i_1_sexe | 0.215 | 0.013 | 16.084 | 0.000 | 0.189 | 0.241 | 0.215 | 1.000 |
| atrsttntts_t2_ | 0.247 | 0.005 | 49.947 | 0.000 | 0.237 | 0.257 | 0.247 | 1.000 |

#### R-Square

|  | Estimate |
| --- | --- |
| rtn_stn1_t1_sm | 0.444 |
| rtn_stn2_t1_sm | 0.731 |
| rtn_stn3_t1_sm | 0.405 |
| rtn_stn4_t1_sm | 0.384 |
| rtn_stn5_t1_sm | 0.327 |
| q5_1.i1._sem | 0.657 |
| q5_1.i2._sem | 0.569 |
| q5_1.i3._sem | 0.396 |
| q5_1.i4._sem | 0.291 |
| q5_1.i5._sem | 0.278 |
| v_2_2_prsnn_r_ | 0.248 |
| v_2_3_frm_str_ | 0.285 |
| v_2_1_snsb_r__ | 0.776 |
| tb_anxieux | 0.102 |
| tb_anxieux_t2 | 0.147 |
| i_5_1_1_chl__2 | 0.087 |
| barrieresT1 | 0.291 |
| barrieresT2 | 0.394 |
